# Comparative *N*-Glycosylation Analysis of the Fc Portions of a Chimeric Human Coagulation Factor VIII and Immunoglobulin G1

**DOI:** 10.3390/bioengineering4020044

**Published:** 2017-05-17

**Authors:** Christoph Kannicht, Mario Kröning, Barbara A. Solecka-Witulska, Guido Kohla, Julia Rosenlöcher

**Affiliations:** Octapharma Biopharmaceuticals GmbH, Molecular Biochemistry, Walther-Nernst-Strasse 3, 12489 Berlin, Germany; mario.kroening@octapharma.com (M.K.); barbara.solecka@octapharma.com (B.A.S.-W.); guido.kohla@octapharma.com (G.K.); julia.rosenloecher@octapharma.com (J.R.)

**Keywords:** Fc-fusion, immunoglobulin G, immunomodulation, *N*-glycosylation, recombinant FVIII

## Abstract

Prevention and treatment of bleeding in patients suffering from hemophilia A are inconvenient due to repeated intravenous infusions owing to the short half-life of coagulation factor VIII (FVIII) in circulation. Besides (glyco-)pegylation of the FVIII molecule, a bioengineering approach comprises the protein fusion to Fc-immunoglobulin (Ig)G that mediate protection from clearance or degradation via binding to the neonatal Fc receptor. While human-like *N*-glycosylation of recombinant FVIII is known to be crucial for the clotting factor’s quality and function, the particular glycosylation of the fused Fc portion has not been investigated in detail so far, despite its known impact on Fcγ receptor binding. Here, we analyzed the *N*-glycosylation of the Fc part of a chimeric FVIII-Fc protein compared to a commercial IgG1 purified from human plasma. Fc parts from both samples were released by enzymatic cleavage and were subsequently separated via sodium dodecyl sulfate polyacrylamide gel electrophoresis (SDS-PAGE). Corresponding protein bands were referred to PNGase F in-gel digestion in order to release the respective *N*-glycans. Analysis via matrix-assisted laser desorption/ionization time-of-flight (MALDI-TOF) mass spectrometry revealed structural differences of both *N*-glycan patterns. Labeling with 2-aminobenzamide (2AB) and analysis via hydrophilic interaction liquid chromatography (HILIC) allowed a quantitative comparison of the respective *N*-glycosylation. Observed variations in Fc glycosylation of the chimeric FVIII fusion protein and human plasma-derived IgG1, e.g., regarding terminal sialylation, are discussed, focusing on the impact of the clotting factor’s properties, most notably its binding to Fcγ receptors.

## 1. Introduction

Hemophilia A is a hereditary blood coagulation disorder caused by the functional deficiency of endogenous clotting factor VIII (FVIII). To achieve a sufficient level of hemostasis, patients are mainly treated by life-long clotting FVIII replacement therapy via on-demand or prophylactic dosing regimens [1]. Efficient prevention and treatment of bleeding in hemophilia A patients requires repeated intravenous infusions. Inconvenience of frequent venipunctures and, particularly in regard to young patients, problems of venous access associated with infections, are critical challenges in hemophilia treatment [2].

In order to reduce the frequency of injections, considerable efforts have been made to prolong the half-life of coagulation factor concentrates. In this regard, main bioengineering strategies include chemical conjugation of polyethylene glycol to FVIII or the generation of recombinant fusion constructs of the clotting factor with human plasma proteins such as serum albumin or immunoglobulin (Ig)G [3]. The latter one is an established technology already applied to several therapeutic protein drugs [4]. It mainly comprises the fusion of the constant fragment crystallizable (Fc) domain of human IgG to a target protein enabling its binding to the neonatal Fc receptor (FcRn) [5] and therefore facilitating the molecule’s recycling. 

Although the immunogenic potential of the Fc fusion technology was ranked to be modest for those fusion constructs currently marked [4], the question of immunogenicity remains unanswered and is particularly critical for FVIII products. While most biotherapeutics induce anti-drug antibodies in <5% of patients, FVIII triggers inhibitory responses in up to 30% of the treated hemophiliacs [3]. The occurrence of neutralizing antibodies, so-called FVIII inhibitors, is a serious clinical challenge associated with an enormous reduction of therapy efficacy, increased morbidity and high additional treatment costs to manage bleeding episodes [6].

Though binding of the IgG-Fc portion to FcRn and related half-life prolongation has been thoroughly discussed, much less attention has been centered on further possible interactions with different receptors. It is known that the antibody’s Fc domain does not exclusively bind to FcRn but also interacts with a set of Fcγ receptors, mediating diverse activating and inhibitory effector activities and, thus regulating immune response [7]. As recently shown, potential immunological consequences of the Fc fusion approach are complex and not yet entirely understood [8,9]. 

One major regulator of Fc-receptor binding affinities is the Fc *N*-glycosylation—a highly versatile post-translational protein modification characterized by covalently attached carbohydrate moieties. Human IgG shares just one highly conserved *N*-glycosylation site at the asparagine residue 297 of the Fc portion, but respective glycoforms are known to crucially influence the effector functions of IgGs [10]. In this regard, the *N*-glycosylation profile of a chimeric FVIII-Fc analog with respect to its Fc fusion part is of particular interest. So far, available literature has just mentioned the occurrence of three main structures that correspond to biantennary oligosaccharides with varying levels of terminal galactose [11]. Here, we have therefore analyzed the Fc *N*-glycosylation of a recombinant FVIII-Fc fusion protein in more detail, drawing qualitative and quantitative comparisons to IgG1 purified from human plasma.

## 2. Materials and Methods

### 2.1. Materials

Human cell line-derived chimeric FVIII-Fc (ELOCTA^®^, Sobi, Stockholm, Sweden) was obtained commercially and dissolved into a protein concentration corresponding to 2000 IU FVIII/mL. IgG1 purified from human plasma was purchased from Athens Research & Technology (Athens, GA, USA). Unless otherwise stated, all chemicals were purchased from Sigma-Aldrich (St. Louis, MO, USA) or Carl Roth (Karlsruhe, Germany). Solvents were of liquid chromatography mass spectrometry (LC-MS) grade quality.

### 2.2. Enzymatic Cleavage and Separation of the Fc Portion

Both protein samples were desalted and re-buffered using illustra NAP-5 columns (GE Healthcare, Little Chalfont, UK) and subsequently concentrated via lyophilization to give a buffer concentration of 50 mM sodium phosphate pH 7.5. According to the manufacturer’s instructions, enzymatic digestion by IdeZ protease (Promega, Madison, WI, USA) was performed for 1 h at 37 °C applying 1 U IdeZ per 1 µg of glycoprotein. Successful cleavage was proven by reducing sodium dodecyl sulfate polyacrylamide gel electrophoresis (SDS-PAGE). Therefore, about 15 µg total protein per lane was separated on a precast 8–16% Tris-glycine gradient gel (anamed Elektrophorese GmbH, Groß-Bieberau, Germany) and subsequently subjected to colloidal Roti^®^-Blue Coomassie (Carl Roth, Karlsruhe, Germany) staining and appropriate destaining in 20% (v/v) ethanol.

### 2.3. In-Gel Deglycosylation

Stained protein bands corresponding to the Fc parts of the chimeric coagulation FVIII and IgG1 were excised from the gel and cut into small cubes (1 × 1 mm^2^ or smaller) using a scalpel. In-gel deglycosylation was performed based on a procedure introduced by Küster et al. [12] and further modified by Weiz et al. [13]. In order to release a sufficient amount of *N*-linked glycans for subsequent analyses, about six protein bands were used per sample, of which two were always treated separately in one reaction vial, up to the final extraction step. In brief, gel pieces were destained in 100 mM ammonium bicarbonate, gradually shrunk with increasing portions of acetonitrile and subsequently dried in a vacuum centrifuge. Dehydrated gel pieces were dissolved in 20 mM sodium bicarbonate pH 7.0 containing 1000 U of PNGase F (New England Biolabs, Ipswich, MA, USA) per reaction vial as well as 8 g/L n-octyl-β-d-glucopyranoside to enhance the solubility and diffusion of the enzyme. Following an incubation at 37 °C overnight, cleaved *N*-glycans were released from the gel matrix. The extraction steps included repetitive incubation in deionized water at 37 °C with intensive shaking at 800 rpm, followed by partial dehydration of the gel pieces in 60% (v/v) acetonitrile, 0.05% (v/v) trifluoroacetic acid and ultrasonication. In order to maximize the yield of *N*-glycans, vacuo-dried gel pieces were frozen at −80 °C and again subjected to the previous extraction procedure. Respectively, extraction phases were collected and pooled per sample.

### 2.4. Neuraminidase Digestion

Extracted and pooled *N*-glycans were desalted on graphite carbon-based HyperSep™ Hypercarb™ solid phase extraction cartridges (Fisher Scientific, Waltham, MA, USA) as described before [14] and were subsequently lyophilized. Each sample was split into two reaction vials, one of which underwent neuraminidase digestion. In a total reaction volume of 20 µL, the digest was performed using 60 U α2-3,6,8,9 Neuraminidase A (New England Biolabs, Ipswich, MA, USA) according to the manufacturer’s instructions for 3 h at 37 °C. Subsequent to another sample desalting on graphitized carbon cartridges, eluted *N*-glycans were referred to derivatization.

### 2.5. N-Glycan Derivatization

#### 2.5.1. Permethylation

Permethylation of carbohydrate samples converts all hydroxyl groups to their methyl esters or methyl ethers, respectively, and therefore stabilizes the labile moieties of terminal sialic acids. Since it also enhances ionization efficiency, it is a common practice prior to mass spectrometric approaches [15]. The derivatization procedure based on an iodomethane reaction followed standard protocols of the solid sodium hydroxide technique and was already published elsewhere [16]. The reaction of iodomethane (Sigma-Aldrich, St. Louis, MO, USA) was stopped by the addition of chloroform and subsequent washing steps with ultrapure water until achieving a neutral pH of the aqueous phase. Permethylated *N*-glycans were dried in a vacuum centrifuge and subjected to mass spectrometric analysis.

#### 2.5.2. Fluorescent Labeling with 2-Amino Benzamide (2-AB)

Since glycans typically show no or low absorbance in both UV and visible light, labeling with a suitable marker, such as 2-AB, is required for quantitative detection. Using reductive amination chemistry, the free reducing end of the released *N*-glycans was derivatized with the fluorescent tag. Therefore, Signal™ 2-AB-*plus* Labeling kit (ProZyme, Hayward, CA, USA) was used according to the specifications of the manufacturer. Subsequent to the incubation period of 1 h at 65 °C, vacuum-driven post-labeling cleanup via GlycoClean S-*plus* cartridges (ProZyme, Hayward, CA, USA) was performed in order to remove excess dye and labeling reagents. Purified glycans were then eluted from the membrane in 1 mL ultrapure water and subsequently freeze-dried in a vacuum centrifuge.

### 2.6. Matrix-Assisted Laser Desorption/Ionization Time-Of-Flight (MALDI-TOF) MS

Dried and permethylated *N*-glycan samples were dissolved in 75% (v/v) acetonitrile in water and co-crystallized on a ground steel target with 10 mg/mL super-dihydroxy-benzoic acid (sDHB) matrix (Sigma-Aldrich, St. Louis, MO, USA) in 10% (v/v) acetonitrile and 1 mM sodium chloride. Covering a mass range from *m/z* 700 to 3500; spectra were recorded in positive-ion reflector mode on an ultraFleXtreme MALDI-TOF/TOF mass spectrometer (Bruker, Bruker Daltonik GmbH, Bremen, Germany) equipped with smartbeam-II™ laser technology. Subsequent data processing and analysis was performed using the associated software modules flexAnalysis (version 3.4, Bruker Daltonik GmbH, Bremen, Germany) and ProteinScape (version 3.1.5, Bruker Daltonik GmbH, Bremen, Germany). In parallel, peak annotation and MS data interpretation was confirmed using GlycoWorkbench software and integrated database (e.g., Carbbank, GlycomeDB) searches [17]. Graphical representation of glycan structures used in this work, based on the symbol nomenclature of the Consortium of Functional Glycomics [18], was also visualized using the above-mentioned GlycoWorkbench tool. A simplified representation of glycan structures utilized brackets if more than one potential position of the respective monosaccharide was emphasized.

### 2.7. Ultra High-Performance Liquid Chromatography (UHPLC)

In order to specify the oligosaccharide profiling obtained by MALDI-TOF mass spectrometry and, moreover, to gain quantitative structural information, 2-AB labeled and purified *N*-glycan samples were separated using UHPLC on an UltiMate™ 3000 system (Thermo Scientific, Waltham, MA, USA) equipped with a fluorescence detector FLD-3100. Dried samples were dissolved in 5 µL of ultrapure water; an aliquot of 1 µL was injected per run. Respectively, fluorescence signals were detected at 420 nm (excitation wavelength of 320 nm). Peaks were integrated automatically according to pre-defined parameters using the Chromeleon™ 7.2 chromatography software.

#### 2.7.1. Hydrophilic Interaction Liquid Chromatography (HILIC)

An ACQUITY UPLC^®^ Glycoprotein BEH Amide column (Waters, Milford, MA, USA) of fully-porous packaging material (300Å pore size, 1.7 µm particle size, 2.1 × 150 mm^2^ dimension) was used to separate aliquots of labeled *N*-glycan samples according to the hydrophilic contributions of the individual constituent monosaccharides. System calibration was performed using a 2-AB labeled dextran calibration standard (Waters, Milford, MA, USA) in order to express the elution in terms of glucose units (GU) allowing comparison to literature data as well as database entries. Keeping a column temperature of 50 °C, a 60-min gradient of acetonitrile and 50 mM ammonium formate pH 4.4 was applied and fluorescence signals were detected. Peak annotation was based on retention times of commercial 2-AB labeled glycan standards (e.g., Glyko^®^ α(2-6) and α(2-3) sialylated biantennary library; ProZyme, Hayward, CA, USA) and their corresponding GU values. In order to confirm single annotations and peak identity, respective UHPLC fractions were collected, derivatized by permethylation (see Section 2.5.1.) and subsequently analyzed via MALDI-TOF mass spectrometry as described above. The sample aliquots that underwent neuraminidase digestion (see Section 2.4.) were also subjected to this HILIC method, thereby allowing for a conclusion to be drawn on the sialylation level of the analyzed *N*-glycan samples. Relative glycan compositions were calculated based on the respective peak areas of the obtained elution profiles.

#### 2.7.2. Mixed-Mode *N*-Glycan Separation

The GlycanPac™ AXH-1 column (Thermo Scientific, Waltham, MA, USA) of porous silica substrate (175 Å pore size, 1.9 µm particle size, 2.1 × 150 mm^2^ dimension) is a dual phase column showing characteristics of a weak anion exchange mode (elution according to charge) and the HILIC mode mentioned above (see Section 2.7.1.). This mixed-mode separation based on charge, size and polarity allows a quantitative analysis of the different glycan charge states (neutral, monosialylated, disialylated etc.) and therefore is a quick option to confirm the sialylation degree of glycan samples. Aliquots of 2-AB labeled *N*-glycan samples were separated at 30 °C applying a 25-min gradient of acetonitrile, 50 mM ammonium formate pH 4.4 and ultrapure water. According to the instructions of the manufacturer, elution windows of the particular glycan charge states were pre-defined using 2-AB labeled glycan standards of model proteins, such as commercial fetuin from fetal calf serum (Sigma-Aldrich, St. Louis, MO, USA) or other in-house controls.

## 3. Results

*N*-glycosylation analysis of a chimeric human FVIII protein was performed with respect to its Fc fusion part and directly compared to the Fc portion *N*-glycan profile of commercial IgG1 purified from human plasma serving as a reference control throughout the whole work.

### 3.1. Separation of the Fc Parts and N-Glycan Release

**Figure 1:**
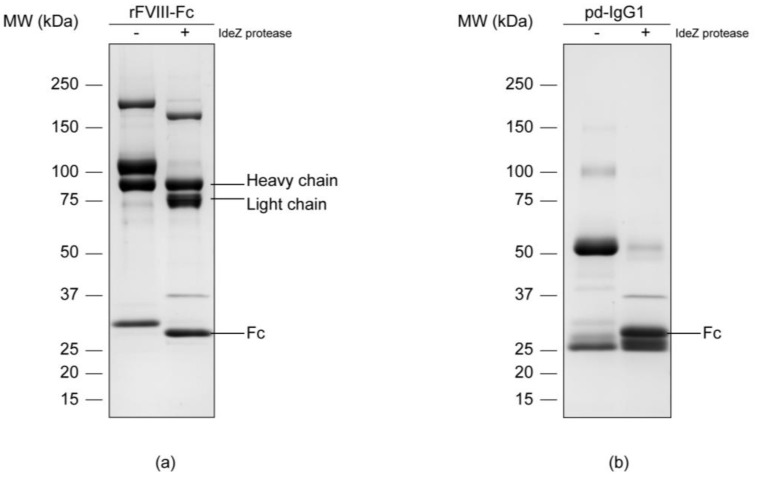
Protein separation of the chimeric human FVIII (**a**) and the plasma-derived IgG1 control (**b**) via reducing SDS-PAGE on an 8–16% Tris-glycine gradient gel with subsequent colloidal Coomassie blue staining. Respectively, the untreated sample (-) was opposed to the IdeZ protease digest (+).

In order to isolate the Fc parts, both samples were subjected to enzymatic digestion with an IdeZ protease that specifically cleaves at a single recognition site next to the IgG hinge region. Successful release was proved via reducing SDS-PAGE separation and subsequent Coomassie blue staining of the respective protein chains (Figure 1). An effective cleavage by IdeZ protease could be observed in the Coomassie-stained gels for both samples. The applied digest conditions as well as the used enzyme amount allowed a nearly complete release of the molecule’s Fc portions. The protease itself gave an additional protein band of approximately 38 kDa which is in accordance with the information provided by the manufacturer.

Protein bands corresponding to the samples’ Fc parts were manually excised from the gel and subsequently underwent in-gel deglycosylation. In this regard, *N*-linked glycans were cleaved by peptide *N*-glycosidase F, commonly known as PNGase F, between the associated asparagine residue and the first *N*-acetylglucosamine (GlcNAc) of the oligosaccharide moiety. Released *N*-glycans were extracted from the gel matrix, desalted and referred to subsequent analyses.

### 3.2. MALDI-TOF Mass Spectrometry

**Figure 2:**
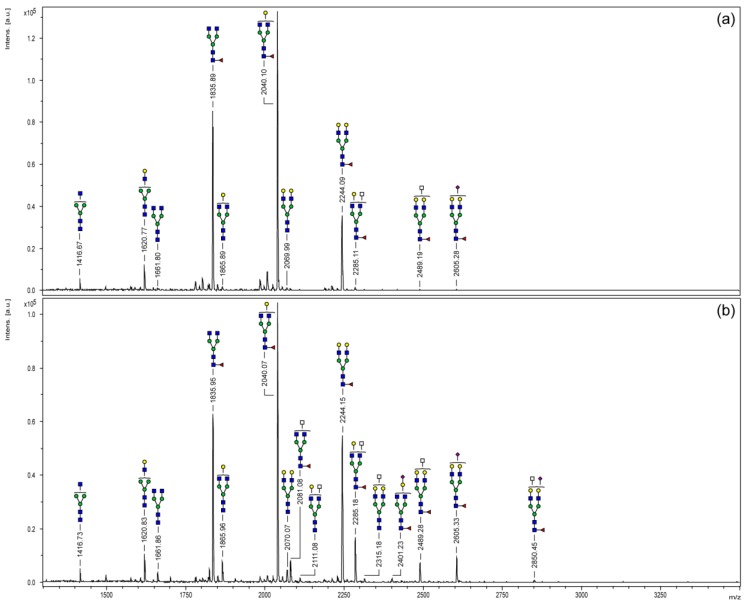
MALDI-TOF mass spectra of permethylated *N*-glycans of the Fc parts of a chimeric FVIII-Fc (**a**) and plasma-derived human IgG1 (**b**). *N*-glycans were released enzymatically via PNGase F in-gel digestion. Molecular ions are present in their sodiated [M + Na]^+^ form.

Purified *N*-glycans were derivatized by permethylation and analyzed via MALDI-TOF mass spectrometry. Figure 2 displays the resulting mass spectra covering a mass range of *m/z* 1100–3500. As commonly known for IgG glycosylation, structural variants were limited to biantennary oligosaccharides. Monofucosylated *N*-glycans bearing none, one or two galactose residues (*m/z* 1835, 2040 and 2244) were the three most abundant signals of both Fc samples. Regarding the Fc part of the chimeric FVIII protein (Figure 2a), a total of 11 different glycoforms have been detected in this study. Only one sialylated *N*-glycan structure (*m/z* 2605) could be observed with minor signal intensity. Since MALDI-TOF analyses provide mass information only, a differentiation between mass-identical *N*-acetylgalactosamine (GalNAc) and GlcNAc or the prediction of a particular monosaccharide position was not possible. However, according to literature, carbohydrate moieties of *m/z* 2285 and 2489 are most likely *N*-glycans bearing a bisecting GlcNAc branch [19,20].

Compared to the chimeric coagulation factor, the Fc part of plasma-derived IgG1 (Figure 2b) showed a slightly more complex *N*-glycosylation profile. A total of 16 glycoforms could be detected. Differences, above all, were observed for the higher mass range (>*m/z* 2100) and were mainly comprised of sialylated structures or carbohydrate moieties bearing an additional *N*-acetylhexosamine (HexNAc; white square) residue.

### 3.3. Chromatographic N-Glycan Profiling

**Figure 3:**
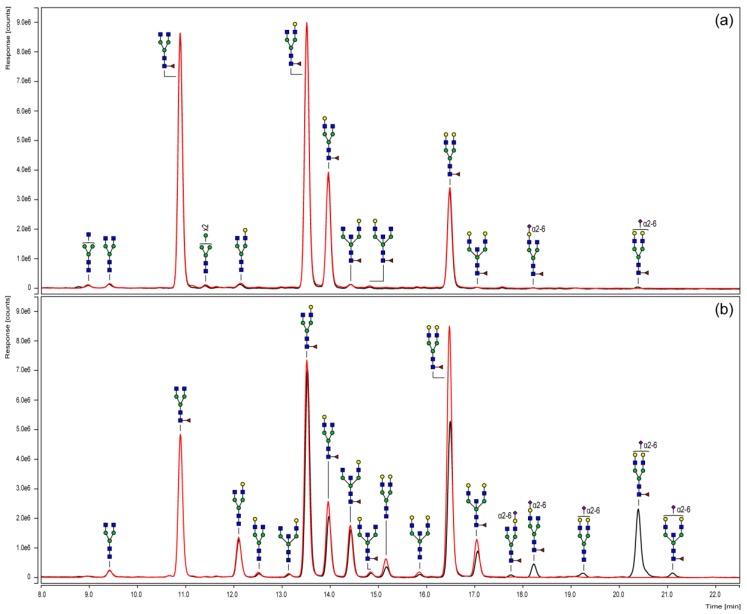
HILIC-UHPLC of 2-AB labeled *N*-glycans derived from in-gel PNGase F treatment of the Fc parts of a chimeric FVIII-Fc fusion protein (**a**) and a plasma-derived IgG1 reference (**b**). The elution profile of a neuraminidase-digested aliquot (red chromatogram) is, respectively, opposed to the untreated sample (black chromatogram).

To elucidate the variations of the Fc *N*-glycosylation of the chimeric coagulation factor and the human IgG1 in more detail and, moreover, in a quantitative manner, UHPLC analyses were performed. For this purpose, *N*-glycans were fluorescently labeled with 2-AB, purified and subsequently separated via HILIC according to their hydrophilicity (Figure 3). In order to specify the sialylation status, sample aliquots also underwent neuraminidase digestion and were compared to their untreated counterparts.

The *N*-glycosylation pattern derived from HILIC-UHPLC basically confirmed the qualitative mass spectrometric results obtained by MALDI-TOF measurements. Furthermore, the chromatographic separation specified the odd HexNAc, as expected, as a bisecting GlcNAc in all considered structures. These analyses also allowed conclusions to be drawn on the sialic acid linkages that, in all cases, were α(2-6)-linked. In addition, position-specific variants, such as those with the galactose residue linked to one or the other antennae, could be separated. 

In addition, neuraminidase cleavage confirmed the identity of sialylated structures. Corresponding peaks shifted to their neutral counterparts and, thus, proved the successful exoglycosidase digest. Regarding the Fc portion of the chimeric coagulation factor (Figure 3a), chromatograms of the digested and untreated sample were almost the same, indicating a negligible level of sialylation. The Fc *N*-glycosylation profile of the plasma-derived IgG reference (Figure 3b) observed a higher portion of sialylated structures, including monosialylated glycoforms with/without fucose or bisecting GlcNAc.

**Figure 4:**
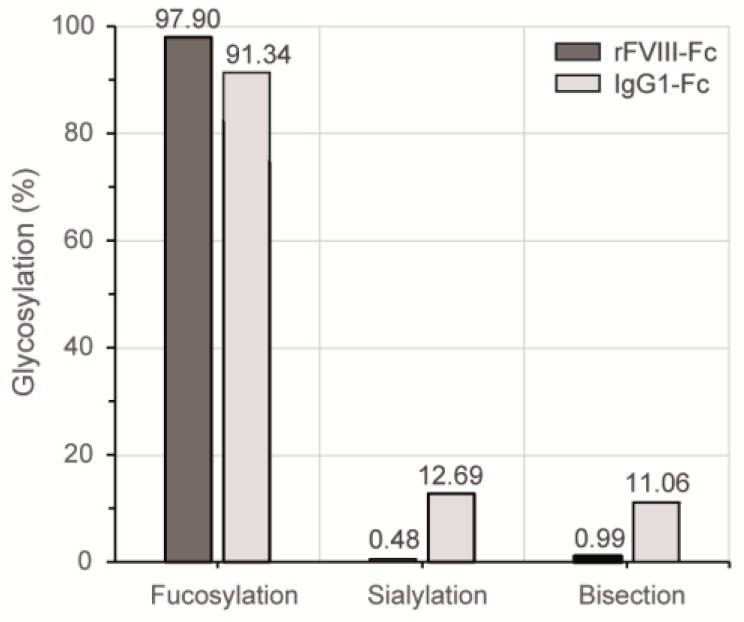
Quantitative comparison of distinct glycosylation attributes of both Fc samples based on their respective HILIC-UHPLC elution profiles shown in Figure 3. Relative distributions were calculated based on the areas of automatically integrated peaks.

The applied 2-AB labeling is a non-selective derivatization procedure and therefore enabled the fluorescence detection of glycans in truly stoichiometric amounts. Quantitative differences of particular glycosylation characteristics between the HILIC elution profiles of both Fc samples are summarized in Figure 4. In comparison to the plasma-derived human IgG1 reference, the level of Fc fucosylation was slightly increased for the chimeric FVIII product, while the most prominent differences were observed regarding sialylation and bisection level. In the case of the recombinant coagulation factor, less than 1% of the detected Fc *N*-glycan structures bore a terminal sialic acid or a bisecting GlcNAc branch.

**Figure 5:**
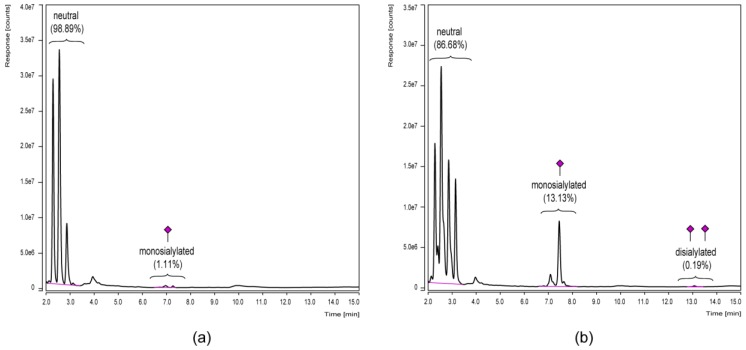
Glycan charge state analysis by UHPLC in a mixed-mode separation of weak anion exchange and HILIC. 2-AB labeled *N*-glycans derived from in-gel PNGase F treatment of the Fc parts of a chimeric FVIII-Fc fusion protein (**a**) and a plasma-derived IgG1 reference (**b**) are compared. Relative distributions were calculated based on the areas of integrated peak groups.

Since especially terminal monosaccharides of Fc glycans, such as sialic acids, are known to affect IgG effector functions [10] and are therefore of special interest, the differential sialylation level was subsequently investigated by an alternative UHPLC approach. In this regard, the mixed-mode separation (weak anion exchange and HILIC) on a dual phase column offered a quick quantitative comparison of the different glycan charge states since they elute in groups of neutral, monosialylated and disialylated variants (Figure 5).

Peak groups of glycan charge states were separated by mixed-mode UHPLC (Figure 5) and therefore visualized the differential sialylation level of the IgG-Fc glycoforms. Portions of sialylated structures were—for both samples—slightly increased compared to the HILIC-UHPLC results presented in Figure 4. Minor discrepancies are explicable since only identified signals were included in the HILIC quantification, while the mixed-mode UHPLC approach was based on peak group integration without the need to identify each single one. However, both approaches clearly showed the increased level of Fc sialylation for the plasma-derived IgG1 reference in contrast to the chimeric FVIII-Fc fusion protein.

## 4. Discussion

Extended half-life FVIII concentrates are one strategy to address the challenges in hemophilia care and to improve the pharmacokinetic protein properties [21]. In 2014, the U.S. Food and Drug Administration granted approval for the first chimeric FVIII-Fc fusion therapeutic. While the desired half-life prolongation effect is primarily based on FcRn binding, the Fc domain also enables interaction with a repertoire of activating and inhibitory Fcγ receptors expressed on various cell types [22]. Regarding recombinant coagulation FVIII, human-like *N*-glycosylation is known to be crucial for the clotting factor’s quality and function [23,24]. In the case of the FVIII-Fc analog, this is assured by recombinant expression in human embryonic kidney cells (HEK-293) providing the human glycosylation machinery [25]. Fc *N*-glycosylation, however, is a major regulator of Fc-receptor binding affinities and associated downstream immunological responses [10]. While total absence of glycosylation is known to disrupt the structural integrity of the Fc region and is therefore required for Fc receptor binding [26], single *N*-glycan monosaccharides have additional impact on the antibody effector functions [10].

Our reported glycan analysis confirmed that Fc glycans of human IgG are mainly of a core-fucosylated complex biantennary type [27]. The analysis revealed a number of structural similarities between Fc *N*-glycan pattern of the chimeric FVIII-Fc fusion protein and the plasma-derived IgG1 reference. However, we could also detect remarkable differences regarding profile complexity, level of bisection and terminal sialylation.

The presence of a bisecting GlcNAc moiety, for instance, has been proven to enhance the binding of Fc to the activating FcγRIIIa in a chemoenzymatic approach, while little effect on the affinity to the inhibitory FcγRIIb could be observed in these binding studies [28]. In this regard, other studies also showed a more potent antibody-dependent cellular cytotoxicity (ADCC) for antibodies, with an increased portion of bisecting GlcNAc residues [29]. However, more recent work stated that just slight changes in bisection have been detected for some antigen-specific IgG responses and little is known so far about their biological relevance [30,31].

The immunomodulatory effect of IgG-Fc core fucosylation, in contrast, is much better understood [10]. The absence of core fucose residues in the Fc glycans substantially increases the ADCC activity of IgG as non-fucosylated antibodies bind to the FcγRIIIa receptor with significantly increased affinity [32]. Our data revealed a high level of core fucosylation for both, the FVIII-Fc protein and human IgG1, suggesting no immunomodulatory activity via enhanced FcγRIIIa receptor binding due to low core fucosylation.

As terminal and the only charged sugar moiety, sialic acid is reviewed to have the main effect on Fc structure [30]. High sialylation level is related to anti-inflammatory effects via the Th2 pathway and decreased ADCC via reduced binding affinity to activating Fcγ receptors [33,34]. Most important, immunosuppressive capacity of IgGs is completely lost upon removal of sialic acid (reviewed in [35]). Here, we observed a sialylation level of ~13% for the human plasma-derived IgG1 reference, which is in close accord with published data for IgG1 [19]. Interestingly, the Fc *N*-glycosylation of chimeric FVIII-Fc revealed only minor sialylation of ≤1%. This degree of sialylation is remarkably low, also in comparison to the known glycan profile of recombinant FVIII without Fc fusion [23]. Data of site-specific FVIII *N*-glycan analysis, likewise referring to a FVIII production in human HEK-293 cells, showed sialylated structures with relative abundance of 37% (Asn 41), 12% (Asn 239) and 16% (Asn 1810) for asparagine residues bearing complex-type *N*-glycans. Thus, the low level of terminal sialic acid seems to be Fc-specific (determined by the protein’s folding and accessibility of the respective *N*-glycosylation sites) rather than a cause of the cell line’s glycosylation potential. The Fc *N*-glycosylation therefore displays almost no immunosuppressive capacity and could have an impact on the immunological potential of the fusion construct.

The immunogenicity of the FVIII-Fc construct has been investigated by Krishnamoorthy and coworkers in a mouse model of hemophilia A compared to a B-domain deleted (BDD) and a full-length rFVIII [36]. In that publication, the authors showed a concentration-dependent immunological effect for chimeric FVIII-Fc in comparison to FVIII without Fc fusion. At low protein concentrations, a less intense immune response was observed for FVIII-Fc, whereas the immune response to FVIII-Fc was higher in contrast to BDD rFVIII at higher concentrations. The reduced immune response to FVIII-Fc at lower doses was discussed to be possibly mediated by the interaction of the Fc-part with the immunosuppressive FcγRIIb receptor. However, the almost complete absence of sialylated *N*-glycans within the Fc portion of FVIII-Fc shown in this work strongly suggests low interaction with the immunosuppressive FcγRIIb receptor.

A different study focusing on the coagulation factor IX-Fc (FIX-Fc) fusion protein described increased immunogenicity of the FIX-Fc in comparison with the native FIX in a hemophilia B mouse model [9]. Moreover, the affinity of the human FIX-Fc manufactured also in a HEK-293 expression system (ALPROLIX^®^, Biogen, Cambridge, MA, USA) to the immunosuppressive FcγRIIb receptor, was determined to be much lower than the affinity to the pro-inflammatory activating FcγRI, FcγRIIa, and FcγRIIIa receptors. However, the glycosylation of the Fc part of the FIX-Fc fusion protein has not yet been investigated and could give further insights. 

Here, we reported for the first time a detailed *N*-glycosylation analysis of recombinant chimeric FVIII with respect to its Fc fusion part. Due to the importance of Fc glycosylation in terms of receptor binding and downstream effector functions, our data supports a better understanding of the immunogenic potential of the highly complex Fc fusion technology that is still not yet fully resolved. The immunomodulatory potential of the Fc fusion platform technology has already been discussed elsewhere [8,9], but we now want to emphasize the importance of Fc glycosylation in this context. Accordingly, targeted glyco-engineering, particularly focused on the Fc region, would be an interesting and worthwhile approach. Methods such as the overexpression of key glycosyltransferases, metabolic glyco-engineering or in vitro techniques were recently described by Dekkers and colleagues in order to generate IgG with defined Fc glycans [31] and could further be applied to chimeric Fc fusion proteins. Regarding chimeric FVIII-Fc, it should also be considered that the coagulation factor in vivo circulates in a close non-covalent complex with its carrier protein von Willebrand factor (vWF) [37]. Since each subunit of multimeric vWF contains one FVIII binding site, multiple FVIII molecules, and consequently multiple clustered Fc domains of the fusion construct, might be presented and may therefore increase associated immunological responses.
